# Infective endocarditis in hypertrophic cardiomyopathy: A multicenter, prospective, cohort study

**DOI:** 10.1097/MD.0000000000004008

**Published:** 2016-07-01

**Authors:** Fernando Dominguez, Antonio Ramos, Emilio Bouza, Patricia Muñoz, Maricela C. Valerio, M. Carmen Fariñas, José Ramón de Berrazueta, Jesús Zarauza, Juan Manuel Pericás Pulido, Juan Carlos Paré, Arístides de Alarcón, Dolores Sousa, Isabel Rodriguez Bailón, Miguel Montejo-Baranda, Mariam Noureddine, Elisa García Vázquez, Pablo Garcia-Pavia

**Affiliations:** a Department of Cardiology, Heart Failure and Inherited Cardiac Diseases Unit, Hospital Universitario Puerta de Hierro; b Infectious Diseases Unit, Department of Internal Medicine, Hospital Universitario Puerta de Hierro; c Clinical Microbiology and Infectious Diseases Unit, Hospital General Universitario Gregorio Marañón; d Health Research Institute Gregorio Marañón, CIBER Respiratory Diseases-CIBERES (CB06/06/0058), Medical School, Complutense University, Madrid; e Infectious Diseases Unit, Hospital Universitario Marqués de Valdecilla; f Department of Cardiology, Hospital Universitario Marqués de Valdecilla; g Department of Cardiology, Hospital de Sierrallana, Santander; h Infectious Diseases Unit, Hospital Clinic-IDIBAPS, Barcelona University; i Department of Cardiology, Hospital Clinic-IDIBAPS, Barcelona University, Barcelona; j Department of Infectious Diseases, Hospital Universitario Virgen del Rocío, Seville; k Infectious Diseases Unit, Complejo Hospitalario Universitario A Coruña, A Coruña; l Department of Cardiology, Hospital Virgen de la Victoria, Málaga; m Infectious Diseases Unit, Hospital Universitario Cruces, País Vasco University, Bilbao; n Department of Internal Medicine, Hospital Costa del Sol, Marbella, Málaga; o Department of Internal Medicine-Infectious Diseases, Hospital Universitario Virgen de la Arrixaca, Medical School, Murcia University, Murcia; p Myocardial Biology Programme, Centro Nacional de Investigaciones Cardiovasculares (CNIC), Madrid, Spain.

**Keywords:** antibiotic prophylaxis, endocarditis, hypertrophic cardiomyopathy

## Abstract

Infective endocarditis (IE) complicating hypertrophic cardiomyopathy (HCM) is a poorly known entity. Although current guidelines do not recommend IE antibiotic prophylaxis (IEAP) in HCM, controversy remains.

This study sought to describe the clinical course of a large series of IE HCM and to compare IE in HCM patients with IE patients with and without an indication for IEAP.

Data from the GAMES IE registry involving 27 Spanish hospitals were analyzed. From January 2008 to December 2013, 2000 consecutive IE patients were prospectively included in the registry. Eleven IE HCM additional cases from before 2008 were also studied. Clinical, microbiological, and echocardiographic characteristics were analyzed in IE HCM patients (n = 34) and in IE HCM reported in literature (n = 84). Patients with nondevice IE (n = 1807) were classified into 3 groups: group 1, HCM with native-valve IE (n = 26); group 2, patients with IEAP indication (n = 696); group 3, patients with no IEAP indication (n = 1085). IE episode and 1-year follow-up data were gathered.

One-year mortality in IE HCM was 42% in our study and 22% in the literature. IE was more frequent, although not exclusive, in obstructive HCM (59% and 74%, respectively). Group 1 exhibited more IE predisposing factors than groups 2 and 3 (62% vs 40% vs 50%, *P* < 0.01), and more previous dental procedures (23% vs 6% vs 8%, *P* < 0.01). Furthermore, Group 1 experienced a higher incidence of *Streptococcus* infections than Group 2 (39% vs 22%, *P* < 0.01) and similar to Group 3 (39% vs 30%, *P* = 0.34). Overall mortality was similar among groups (42% vs 36% vs 35%, *P* = 0.64).

IE occurs in HCM patients with and without obstruction. Mortality of IE HCM is high but similar to patients with and without IEAP indication. Predisposing factors, previous dental procedures, and streptococcal infection are higher in IE HCM, suggesting that HCM patients could benefit from IEAP.

## Abstract 2

Supplemental Digital Content is available in the text

## 1 Introduction

Infective endocarditis (IE) is a recognized complication of hypertrophic cardiomyopathy (HCM). Although IE in HCM has been known for many years, information in the literature is limited to isolated case reports and small (≤11 individuals) case series.^[1,2]^ The incidence of IE among HCM patients has been described to be 18 to 28 times higher than in the general population and left ventricular outflow tract obstruction (LVOTO) and enlarged left atria have been reported as factors that increase the risk of IE in HCM.^[1]^

Until 2007, IE antibiotic prophylaxis (IEAP) was recommended for all HCM patients before invasive procedures, and especially for HCM patients with LVOTO.^[3,4]^ However, in 2007, the American Heart Association (AHA) revised the IEAP recommendations and retired IEAP for HCM patients due to an apparently significant morbidity associated with IEAP therapy, and a lack of evidence supporting efficacy of IEAP in IE prevention.^[5,6]^ This controversial decision has received criticism as it relies on limited scientific evidence, and IE in HCM usually is a very serious complication.^[6]^ Moreover, the 2007 AHA^[5]^ and 2015 ESC^[7]^ revised recommendations for antimicrobial prevention of IE maintained IEAP for several cardiac conditions in which IE might have a similar mortality rate to HCM.

The purpose of this study was 2-fold: to describe the clinical, microbiological, and echocardiographic characteristics in a large series of HCM patients complicated by IE, and to compare the characteristics of IE HCM patients with those of IE patients with and without an indication for IEAP.

## 2 Methods

From January 2008 to December 2013, 2000 consecutive patients with confirmed or possible IE according to the modified Duke criteria^[8]^ were prospectively included in the “Spanish Collaboration on Endocarditis-Grupo de Apoyo al Manejo de la Endocarditis infecciosa en ESpaña (GAMES)” registry at 27 Spanish hospitals. Multidisciplinary teams completed a standardized case report document with IE episode and 1-year follow-up data. Regional and local ethics committees approved the study and patients gave their informed consent.

All data from patients included in this study were retrieved from a standardized case report form that included clinical, microbiological, and echocardiographic sections.

HCM was defined according to current guidelines.^[9]^ LVOTO was defined as a peak instantaneous Doppler LV outflow tract pressure gradient of ≥30 mm Hg at rest or exercise.^[9]^ Prosthetic IE was considered when IE occurred in parts of valve prosthesis (biological or mechanical) or on reconstructed native heart valves. Native-valve IE was considered when IE occurred in a nonoperated native heart valve. Device-related IE was defined as endocarditis affecting a pacemaker or an internal cardiac defibrillator intracardiac lead.

IEAP indications were based on current AHA/ESC recommendations.^[5,7]^ Hence, patients with previous IE, prosthetic valves, unrepaired cyanotic congenital heart disease (CHD), repaired CHD with residual defects, and patients with CHD and <6 months since surgery were considered as candidates for IEAP. Indications for surgery were based on ESC recommendations.^[10]^

To expand the IE HCM group, available prospective local IE databases from participating hospitals were also evaluated for HCM patients who had IE previous to January 2008. Four of the 27 participating hospitals had prospective local IE databases prior to January 2008. Eleven IE HCM additional cases (9 nondevice IE) were identified in these databases and clinical data were gathered using the GAMES standardized case report document. The final study cohort comprised 2011 individuals with IE and the final total number of IE HCM patients was 34, which included 4 patients with device-related IE and 4 with prosthetic valve IE. Therefore, the total number of native-valve IE in HCM patients was 26.

**Figure 1:**
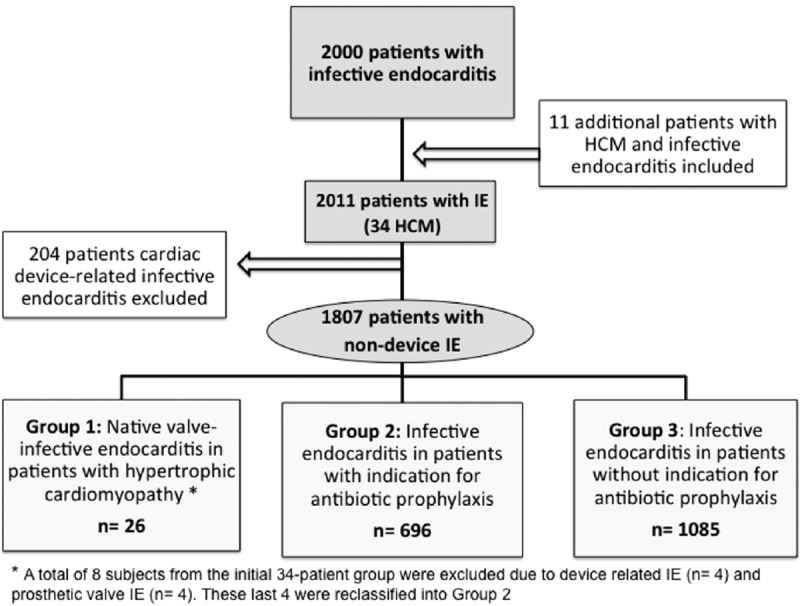
Study flowchart showing patients’ selection process.

In addition, patients with nondevice IE were selected (n = 1807) and were classified into 3 groups: group 1, HCM patients with native-valve IE (n = 26); group 2, patients with IEAP indication (n = 696); group 3, patients without IEAP indication (n = 1085). Four HCM patients had prosthetic valves and were reclassified into group 2. The study selection process is shown in Fig. 1.

### 2.1 Review of literature

For study selection, PubMed and Web of Knowledge electronic databases were searched using the terms “infective endocarditis” and “hypertrophic cardiomyopathy” in the title and abstract (Fig. S1 of supplementary material). The last search was performed on May 1, 2014. Papers were eligible if they described IE complicating HCM, limited to English and Spanish languages.

### 2.2 Statistical analysis

Results are presented as mean (standard deviation) for continuous variables with normal distribution, as median (interquartile range) for continuous variables without normal distribution, and as number (percentage) for categorical data. For statistical analysis, Student *t* test and Mann-Whitney nonparametric test were used in 2-group comparisons, whereas analysis of variance and Tukey test for multiple group comparisons were applied for 3 groups. Chi-square test or Fisher exact test were used for categorical variables.

A 2-tailed *P* < 0.05 was considered statistically significant. The entire analysis was performed using the SPSS package, version 16.0 (SPSS Inc, Chicago, IL).

## 3 Results

A total of 2011 patients with definite (n = 1653, 82%) or possible (n = 358, 18%) IE were included in the study. The median age of the patients was 69 years (IQR 57–76), and 68% were male. Overall in-hospital and 1-year mortality rate was 27% and 34%, respectively. The baseline characteristics are summarized in Table S1 of supplementary material.

### 3.1 Infective endocarditis in HCM

**Figure 2:**
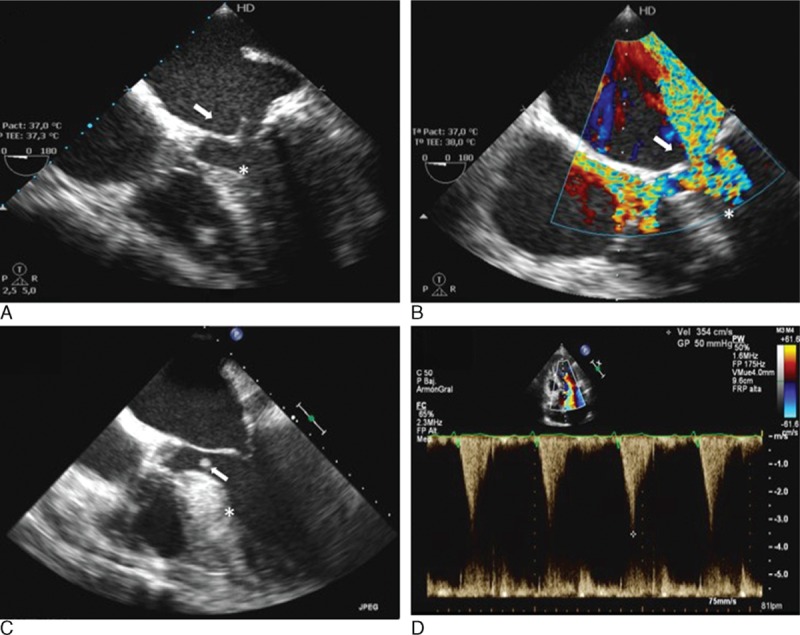
Examples of IE in 2 HCM patients. A and B, Mitral valve endocarditis. A, Transesophageal echocardiogram, 4 chamber view, 0°. An oscillating 16-mm vegetation is observed on the left atrial side of the anterior mitral leaflet (white arrow). Septal hypertrophy of 16 mm (white asterisk). B, Transesophageal echocardiogram, 5 chamber view, 0°. Color Doppler across the mitral valve with evidence of severe mitral regurgitation (white arrow), as well as flow acceleration noted in the left ventricle outflow tract (white asterisk). C and D, Infective endocarditis affecting the basal interventricular septum. C, Transesophageal echocardiogram, 4 chamber view, 0°. An 8- by 4-mm vegetation is evidenced 20 mm below the aortic valve (white arrow). Severe septal hypertrophy with a maximal wall thickness of 27 mm (white asterisk). D, Pulsed wave Doppler at the left ventricular outflow tract. Maximum gradient of 50 mm Hg and peak velocity of 3.5 m/s, with the characteristic dagger-shaped appearance seen in obstructive HCM. HCM = hypertrophic cardiomyopathy, IE = infective endocarditis.

Among the 27 hospitals of the GAMES registry, 34 HCM patients with IE (4 patients with device-related IE and 4 with prosthetic valve IE) from 13 different centers were identified. Median age was 64 (IQR 57–74), and 56% were male. Distribution of hypertrophy was predominantly concentric (38%) and septal (29%). The mitral valve was the most frequently affected valve (n = 24, 71%), either in isolation (75%) or combined with other affected valves (25%) (Fig. 2). LVOTO was present in 19 patients (56%).

The clinical, echocardiographic and microbiological characteristics of HCM patients with IE are shown in Table 1. A high proportion of HCM patients had a suspected predisposing factor for bacteremia (n = 22, 65%). The oral cavity was the main supposed source of infection (18% of patients) and *Streptococcus* spp. was the most frequent infective agent identified (35%).

**Table 1:**
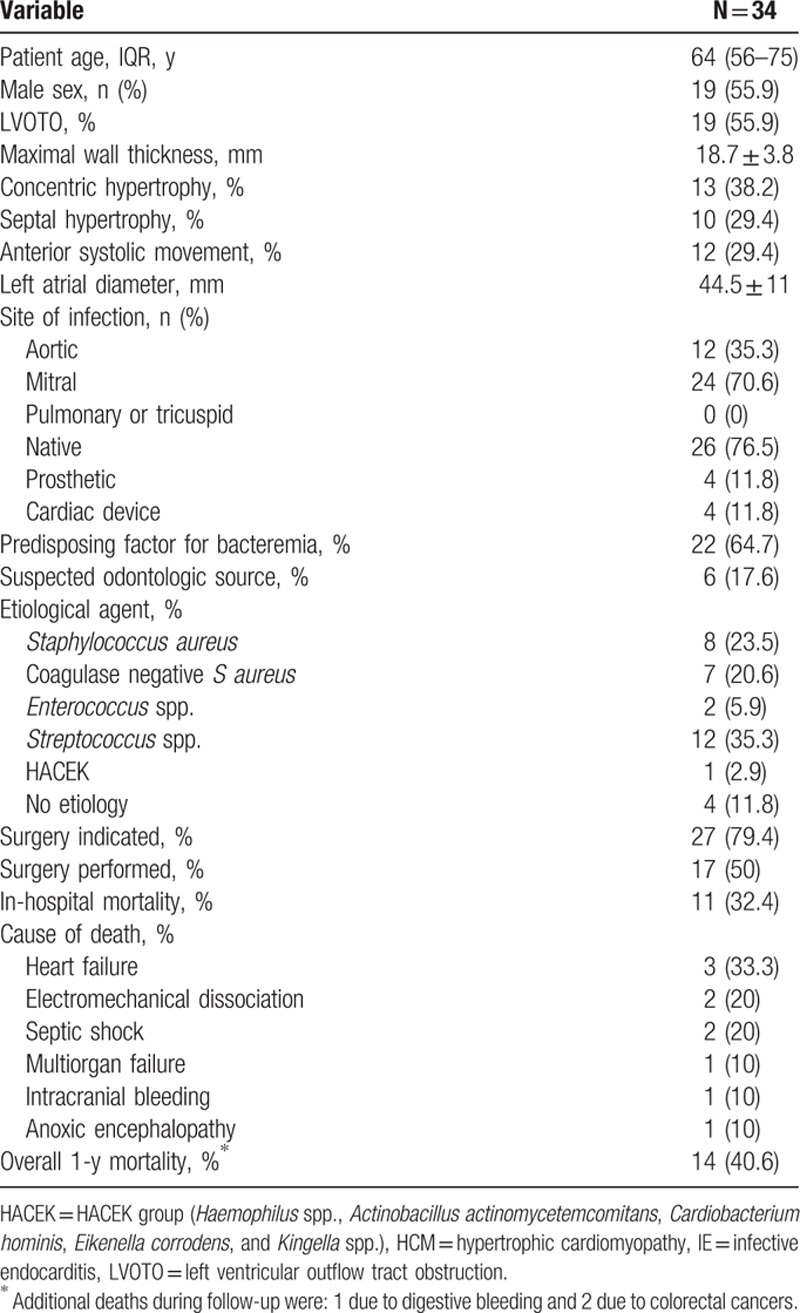
Clinical, echocardiographic, and microbiological characteristics of 34 HCM patients with IE.

Regarding clinical complications, 7 patients suffered a stroke (21%) and 9 patients had peripheral embolisms (27%). Surgery was conducted in 17 patients (50%).

Eleven patients (32%) died before hospital discharge and overall 1-year mortality was 41%. Causes of death are shown in Table 1. LVOTO was not related with a higher morbidity or mortality (data not shown). Indeed, intracardiac complications were more common in the non-LVOTO group (63% vs 21%, *P* = 0.037).

### 3.2 Review of literature

From 1961 to May 2014, a total of 84 cases of IE complicating HCM have been described in the literature within a total of 47 records (Fig. S1)^1,2,w1-w45^. In addition, 3 letters to the editor concerning this topic have been published during the aforementioned period of time.^[6,11,12]^

Patients’ clinical, microbiological, and echocardiographic characteristics are summarized in Table S2 of supplementary material. Whenever available, echocardiography has played a fundamental role in diagnosis, although clinical and microbiological findings were occasionally the only diagnostic tools in the initial case reports. Overall, there was a predominance of male sex (59%), and mean age was 47.3 (16.3) years. The mitral valve was the most frequently affected valve, including multisite infections (59%). Contrary to what has been stated in previous reports,^[1]^ not all cases of IE complicating HCM showed LVOTO, but its prevalence was very high (74%). The most frequent infective agent was *Streptococcus* spp. (40% of cases). Predisposing factors for IE were present in 43% of patients, and dental procedures were the most frequent among them (47%). Surgery was globally performed in 43% of patients. However, surgical procedures significantly increased after 1990, 54% vs 27% in the previous period (*P* = 0.04).

Overall mortality of IE HCM cases published in the period 1961 to 2014 was 22%. Mortality rate of cases published after 1990 was 14%, compared with 33% in cases published before 1990 (*P* = 0.05). Patients with LVOTO exhibited a higher, but not significant, mortality rate than patients without LVOTO (26% vs 8%, *P* = 0.42).

### 3.3 Infective endocarditis in HCM compared with infective endocarditis in IEAP groups

A total of 1807 patients (67% males; median age 68 years [IQR 56–76]) had definite (n = 1505, 83%) or possible (n = 302, 17%) nondevice IE. Overall in-hospital and 1-year mortality rate was 29% and 36%, respectively. The baseline characteristics are summarized in Table S3 of supplementary material.

#### 3.3.1 Group 1 vs group 2

No differences between native-valve IE HCM patients (n = 26) and patients with IE with IEAP indication (n = 696) were observed in terms of age, sex, and ejection fraction. Among HCM patients, there was more IE affecting the mitral valve, (85% vs 42%, *P* < 0.01) as well as a higher proportion of patients with mitral regurgitation and congestive heart failure at diagnosis (Table 2). A predisposing factor for IE was found more frequently in patients with HCM than in patients with IEAP indication (62% vs 40%, *P* < 0.02). More specifically, a dental origin of infection was described more frequently in the HCM group (23% vs 6%, *P* = 0.02). No differences were found regarding previous genitourinary (0% vs 3.5%, *P* = 0.36), cutaneous (9.1% vs 4.4%, *P* = 0.32), vascular (19.2% vs 16.1%, *P* = 0.68), gastrointestinal (11.5% vs 6.1%, *P* = 0.26), or respiratory (0% vs 0.6%, *P* = 0.27) procedures.

**Table 2:**
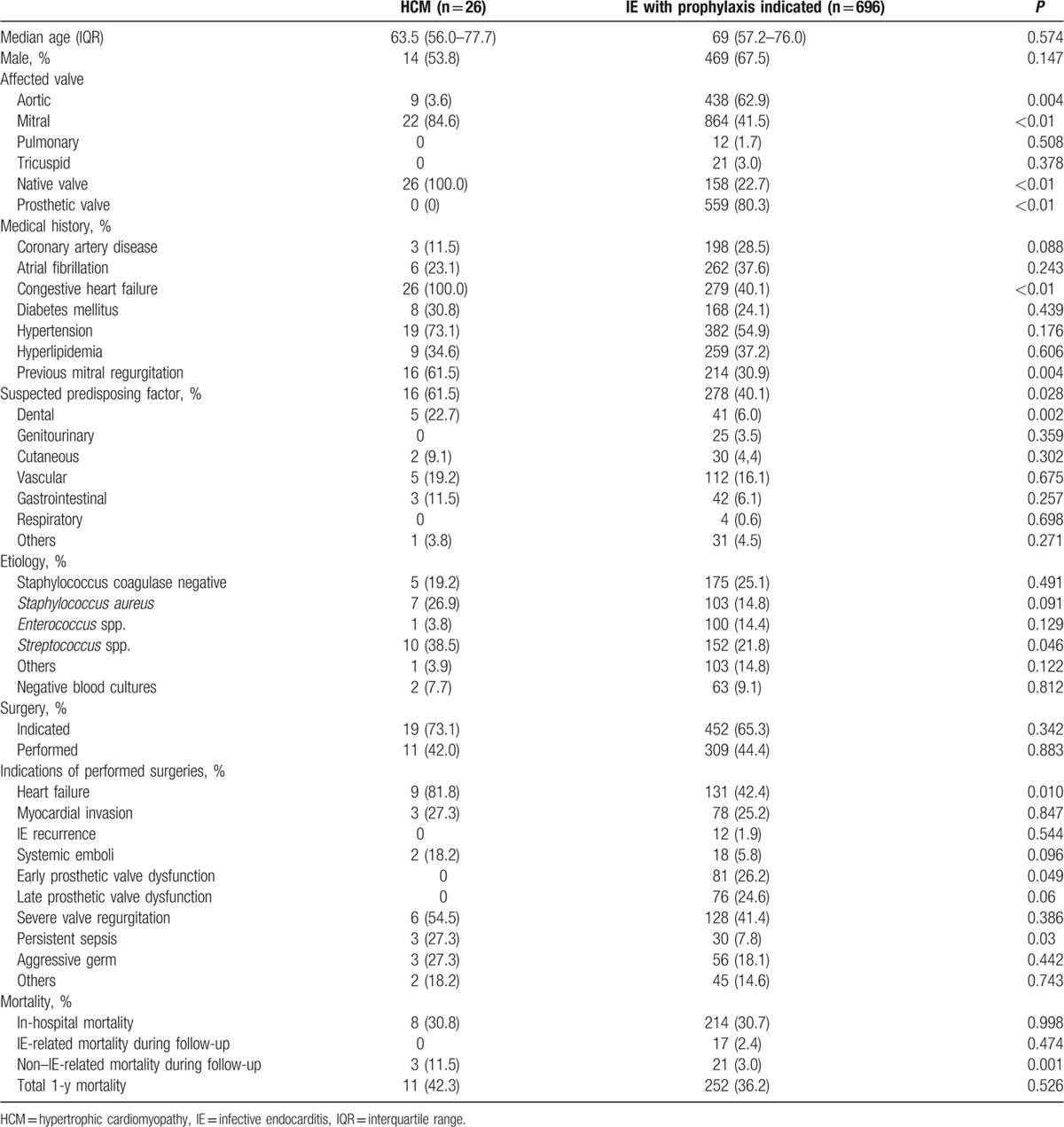
IE in HCM patients (group 1) compared with IE patients with indication for IE antibiotic prophylaxis (group 2).

The main infective agent causing IE in HCM patients was *Streptococcus* spp., with an incidence almost twice as high to that found in patients from group 2 (39% vs 22%, *P* < 0.05) (Table 2). No differences were found between the groups in the incidence of other common bacterial agents such as *Staphylococcus* and *Enterococcus*. The proportion of patients in whom surgery was indicated attending to IE guidelines was similar in both groups, but a higher percentage required surgery because of heart failure in the HCM group (82% vs 42%, *P* = 0.01). Furthermore, persistent sepsis was more frequent as an indication for surgery in the HCM group (27% vs 8%, *P* = 0.03). No significant differences between groups were observed in relation to in-hospital and 1-year mortality (Table 2).

#### 3.3.2 Group 1 vs group 3

Age, sex distribution, ejection fraction, and cardiovascular risk factors were similar between both groups (Table 3). Mitral valve IE was significantly higher in the HCM group than in IE patients without IEAP indication (85% vs 53%, *P* = 0.001). At diagnosis, heart failure signs were present in all IE HCM patients. IE of presumed dental origin was again higher in HCM patients (23% vs 8%, *P* = 0.009). There were no differences regarding previous genitourinary (0% vs 7%, *P* = 0.2), cutaneous (9.1% vs 7.4%, *P* = 0.77), vascular (19.2% vs 16.8%, *P* = 0.74), gastrointestinal (11.5% vs 8.4%, *P* = 0.57), or respiratory (0% vs 1.6%, *P* = 0.52) procedures. There was a strong tendency toward more surgery performed because of uncontrolled infection in the HCM group (27% vs 7%, *P* = 0.016); however, infective agents were similar in both groups.

**Table 3:**
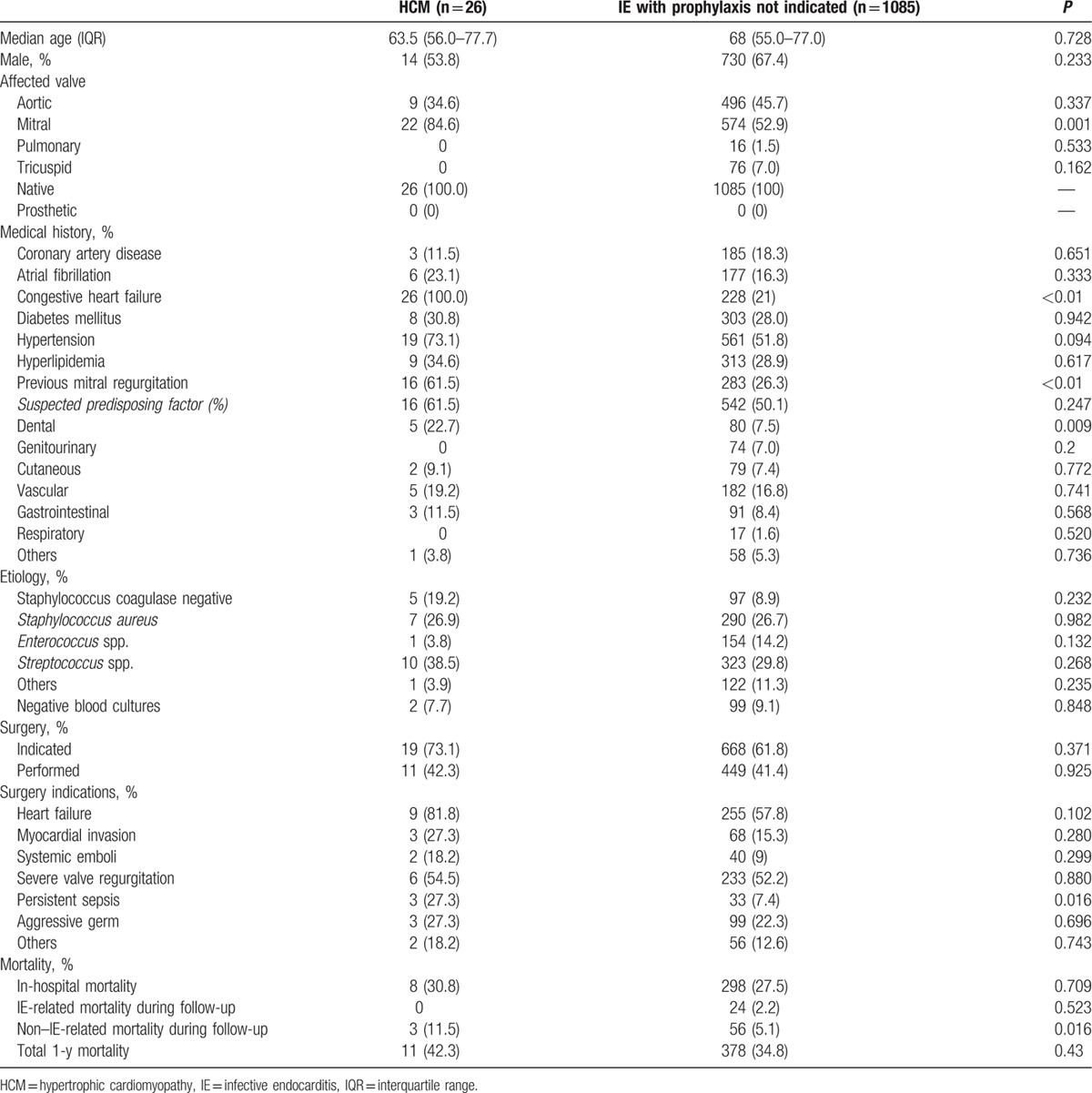
IE in HCM patients (group 1) compared with IE patients without indication for antibiotic prophylaxis (group 3).

IE mortality during hospital stay or follow-up did not differ between both groups. Nonetheless, mortality related to other causes was higher in the HCM group (Table 3).

## 4 Discussion

Infective endocarditis in patients with HCM is a serious complication associated with high morbidity and mortality.^[2]^ Available information is confined to case reports and very small case series^1,2,w1-w45^. Moreover, controversy exists in the field regarding whether HCM patients should receive antibiotic prophylaxis before dental procedures to prevent IE.^[6,13]^ This study is the largest cohort of IE HCM reported to date and provides an updated view of this complication through a multicenter collaboration of nonspecialized and specialized centers. In addition, the comparison of clinical characteristics of native-valve IE HCM patients with the findings of 1781 infective valve endocarditis patients classified according to their indication for IEAP provides important insight into the potential usefulness of IEAP in HCM individuals.

### 4.1 Clinical features of HCM patients with IE

The mitral valve was the most affected valve in our study (71%), which is consistent with previous descriptions in the literature. The long mitral leaflets seen in HCM patients^[14]^ and the centrifugal effect of the turbulent flow in the left ventricular outflow tract present in up to two thirds of HCM individuals^[15]^ render these patients more susceptible to IE due to erosion of the endocardium layer. Interestingly, we found that up to 44% of HCM patients developed IE without LVOTO, which contrasts with some previous studies in which all IE HCM patients had LVOTO.^[1]^ In addition, we did not find clinical differences between IE HCM patients with or without obstruction.

Regarding predisposing factors for IE, we found a higher prevalence of suspected sources for bacteremia in patients with HCM, versus both patients with and without IEAP indication. Previous case reports also have described predisposing factors in a high proportion of patients with HCM who developed IE as shown in Table S2 from supplementary material. It could have been expected that HCM patients would have had a higher prevalence of suspected sources for bacteremia than patients with IEAP indication, but this difference was also observed when HCM patients were compared with patients without IEAP indication in relation to previous dental procedures. Therefore, patients with HCM might have more risk of developing IE after hematogenous spread of bacteria from the oral cavity than the general population.

In addition, persistent sepsis was more common among IE HCM patients compared with the other 2 groups. HCM patients usually exhibit increased intracardiac pressures and have higher prevalence of elongated mitral leaflets that favor sustained erosion, which facilitates the settling of microorganisms in the heart valves, and they may not adequately respond to antibiotic treatment. Thus, it would be possible that IE in HCM results in an increased risk for developing heart failure and persistent sepsis.

*Streptococcus* spp. was the most frequent causative agent for IE in our cohort of HCM patients, as it was in almost 40% of the previously published case reports. Assuming that *Streptococci* are the most prevalent species in the oral cavity^[16]^ and that HCM patients are not recommended to receive IEAP, we found that these microorganisms were more prevalent in native-valve IE HCM patients than in patients with IEAP indication, but similar to patients without IEAP indication. However, while the microbiological spectrum was similar in native-valve IE HCM compared with this last group, native-valve IE HCM patients showed higher morbidity.

Although 79% of IE HCM patients had indications for surgery, only 50% were finally operated on. This percentage is similar to what is found in the literature from 1961 to 2014 but lower if more recent cases are considered (Table S2). A possible explanation for this is the higher surgical risk of HCM patients, which are hemodynamically more difficult to manage for both surgeons and anesthesiologists, and also the fact that our cohort comprises 34 IE HCM patients from 13 hospitals with very different complexity levels.

The overall mortality of the published case reports and case series for the period 1961 to 2014 was 22%. This is strikingly lower than that observed in our series (32% in-hospital and 41% after 1 year). One possible explanation for this difference is that the published cases mainly describe in-hospital mortality. Moreover, considering that most of the literature refers to single patient case reports (Table S2), a publication bias might be present as cases with poor outcome are less likely to be communicated. Furthermore, our series reflects results from a nationwide prospective registry including nonspecialized centers. Therefore, we consider that the real-world mortality of IE HCM patients would likely be more similar to that found in our study than that currently available from the literature.

Although IE is uncommon within the global HCM population,^[1]^ HCM patients have 18 to 28 times more risk of IE than the general population and the risk in obstructive HCM is 48 to 80 times higher (estimated incidence of IE in HCM and obstructive HCM is 1.4 and 3.8 per 1000 person-years,^[1]^ vs 5.0 to 7.9 cases per 100,000 person-years in the general population).^[17]^ Moreover, when IE occurs in HCM the prognosis is considered to be poor.^[1,2]^ In our cohort of HCM patients with IE, in-hospital mortality was almost identical to that of patients with IEAP indication (30.8% vs 30.7%, *P* = 0.98), as could have been expected assuming that both groups of patients have underlying cardiac disease. Interestingly, when compared with the group of patients without IEAP indication, no statistically significant differences were found in the in-hospital mortality between both groups, although mortality was slightly higher among HCM patients (30.8% vs 27.5%, *P* = 0.7).

### 4.2 HCM and indications for IEAP

Since 2007, the AHA does not recommend IEAP for HCM patients,^[3]^ as it was considered that HCM is not one of the underlying cardiac conditions with a high risk for IE even though data regarding this issue are scarce. Some studies state that IE is more likely to result from frequent exposure to random bacteremia associated with daily activities than from dental, gastrointestinal, or genitourinary procedures.^[18]^ However, animal studies have proven the effectiveness of antibiotics in preventing streptococcal endocarditis after inoculation of bacteria.^[19]^

In our study, previous invasive procedures as predisposing factors for IE were significantly higher among native-valve IE HCM patients than in the group of patients who presumably received IEAP, but similar to the group of patients who did not have IEAP indication. When considering only dental procedures, they were found more frequently among the native-valve IE HCM group than in both with and without IEAP groups. Furthermore, *Streptococci* IE was more prevalent among native-valve IE HCM subjects and patients without IEAP indication, supporting the concept that IEAP can have some effect on the prevention of IE caused by dental-origin bacteremia. Moreover, recent published data from the UK and USA have shown a significant rise in the incidence of total and *Streptococci* IE after IEAP was prohibited/restricted.^[20,21]^

Considering our findings together with the facts that anaphylactic shock secondary to IEAP occurs very rarely (15–25 individuals per 1 million patients who receive a dose of penicillin),^[3]^ no deaths have been reported in HCM patients receiving IEAP so far,^[6]^ and that HCM patients are at increased risk of IE,^[1]^ it seems appropriate to reconsider the balance between benefits and risks of IEAP administration in HCM patients, at least before dental procedures.

### 4.3 Limitations

The IE cohort of the GAMES registry was not specifically designed to address the questions investigated in our study. In particular, whether if patients had received IEAP prior to invasive procedures was not included in the registry and therefore we do not know if native-valve IE HCM patients or patients included in the other groups received IEAP or not. Moreover, 9 native-valve IE HCM patients included in our study had IE prior to 2008 when IEAP was advocated in HCM.

As we have only included patients with IE diagnosis, we do not know the total number of HCM patients among our population, so we cannot provide incidence or prevalence data in this study. Nonetheless, this study provides the largest cohort of IE in HCM patients reported to date.

## 5 Conclusions

Infective endocarditis is an uncommon but serious complication in HCM that can occur in patients with or without LVOTO. Mortality is high but similar to that found in patients with or without indication for endocarditis prophylaxis. However, predisposing factors and *Streptococci* infections are more frequent among native-valve HCM patients, suggesting that these individuals may benefit from IEAP before interventions in the oral cavity.

## Supplementary Material

Supplemental Digital Content
