# Supplementary material for: Infective endocarditis in hypertrophic cardiomyopathy: A multicenter, prospective, cohort study
Source: Medicine (Baltimore). 2016 Jul 1;95(26):e4008. doi: 10.1097/MD.0000000000004008 (PMC4937928; doi:10.1097/MD.0000000000004008)
Supplement: Supplemental Digital Content [file medi-95-e4008-s001.doc]

Figure S1 Study selection process for literature review.


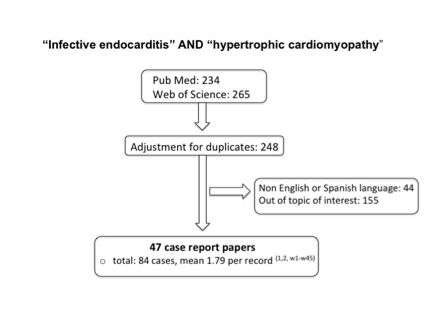


**Appendix**

**Members of GAMES:**

**Hospital Costa del Sol**, (Marbella): Fernando Fernández Sánchez, Mariam Noureddine, Gabriel Rosas, Javier de la Torre Lima; **Hospital Universitario de Cruces**, (Bilbao): José Aramendi, Elena Bereciartua, María Victoria Boado, Marta Campaña Lázaro, Josune Goikoetxea, Juan José Goiti, José Luis Hernández, José Ramón Iruretagoyena, Josu Irurzun Zuazabal, Leire López-Soria, Miguel Montejo, Pedro María Pérez, Regino Rodríguez, Roberto Voces; **Hospital Universitario Virgen de la Victoria**, (Málaga): Mª Victoria García López, Radka Ivanova Georgieva, Manuel Márquez Solero, Isabel Rodríguez Bailón, Josefa Ruiz Morales; **Hospital Universitario Donostia-Policlínica Gipuzkoa**, (San Sebastián): Ana María Cuende, Tomás Echeverría, Ana Fuerte, Eduardo Gaminde, Miguel Ángel Goenaga, Pedro Idígoras, José Antonio Iribarren, Alberto Izaguirre Yarza, Carlos Reviejo; **Hospital General Universitario de Alicante**, (Alicante): Rafael Carrasco, Vicente Climent, Patricio Llamas, Esperanza Merino, Joaquín Plazas, Sergio Reus; **Complejo Hospitalario Universitario A Coruña**, (A Coruña): Nemesio Álvarez, José María Bravo-Ferrer, María del Mar Carmona, Laura Castelo, José Cuenca, Pedro Llinares, Enrique Miguez Rey, María Rodríguez Mayo, Dolores Sousa, Mª Carmen Zúñiga; **Complejo Hospitalario de Especialidades Juan Ramón Jiménez**, (Huelva): Francisco Javier Martínez; **Hospital Universitario de Canarias**, (Canarias): Mª del Mar Alonso, Beatriz Castro, Dácil García Marrero, Mª del Carmen Durán, Mª Antonia Miguel Gómez, Juan La Calzada, Ibrahim Nassar; **Hospital Regional Universitario Carlos Haya**, (Málaga): Antonio Plata Ciezar, José Mª Reguera Iglesias; **Hospital Universitario Central Asturias**, (Oviedo): Víctor Asensi Álvarez, Carlos Costas, Jesús de la Hera, Jonnathan Fernández Suárez, Lisardo Iglesias Fraile, Víctor León Arguero, José López Menéndez, Pilar Mencia Bajo, Carlos Morales, Alfonso Moreno Torrico, Carmen Palomo, Begoña Paya Martínez, Ángeles Rodríguez Esteban, Raquel Rodríguez García, Mauricio Telenti Asensio; **Hospital Universitario Clínic de Barcelona**, (Barcelona): Manuel Almela, Yolanda Armero, Manuel Azqueta, Mercé Brunet, Ramón Cartañá, Carlos Cervera, Carlos Falces, Guillermina Fita, David Fuster, Cristina García de la Maria, José M. Gatell, Jaume Llopis Pérez, Francesc Marco, Carlos A. Mestres, José Mª Miró, Asunción Moreno, Salvador Ninot, Eduardo Quintana, , Carlos Paré, Juan Manuel Pericás, José L. Pomar, José Ramírez, Irene Rovira, Marta Sitges, Dolors Soy, Adrián Téllez, Jordi Vila; **Hospital General Universitario Gregorio Marañón**, (Madrid): Javier Bermejo, Emilio Bouza, Gregorio Cuerpo, Viviana de Egea, Alia Eworo, Ana Fernández Cruz, Mª Eugenia García Leoni, Marcela González del Vecchio, Víctor González Ramallo, Martha Kestler Hernández, Mercedes Marín, Manuel Martínez-Sellés, Mª Cruz Menárguez, Patricia Muñoz, Cristina Rincón, Hugo Rodríguez-Abella, Marta Rodríguez-Créixems, Blanca Pinilla, Ángel Pinto, Maricela Valerio, Eduardo Verde Moreno; **Hospital Universitario La Paz**, (Madrid): Isabel Antorrena, Mar Moreno, José Ramón Paño, Sandra Rosillo, María Romero, Araceli Saldaña; **Hospital Universitario Marqués de Valdecilla**, (Santander): Carlos Armiñanzas Castillo, Ana Arnaiz, José Berrazueta, Sara Bellisco, Manuel Cobo Belaustegui, Raquel Durán, MªCarmen Fariñas, Concepción Fariñas-Álvarez, Carlos Fernández Mazarrasa, Rubén Gómez Izquierdo, Claudia González Rico, José Gutiérrez Díez, Rafael Martín Durán, Marcos Pajarón, José Antonio Parra, Ramón Teira, Jesús Zarauza; **Hospital** **Universitario Puerta de Hierro**, (Madrid): Pablo García-Pavía, Jesús G. Mirelis, Beatriz Orden, Antonio Ramos, Isabel Sanchez, Fernando Dominguez, Carlos Garcia-Montero, Luis Alonso-Pulpón; **Hospital Universitario Ramón y Cajal**, (Madrid): Tomasa Centella, José Manuel Hermida, José Luis Moya, Pilar Martín-Dávila, Enrique Navas, Enrique Oliva, Alejandro del Río, Soledad Ruiz; **Hospital Universitario Virgen de las Nieves**, (Granada): Carmen Hidalgo Tenorio; **Hospital Universitario Virgen Macarena**, (Sevilla): Antonio de Castro, Marina de Cueto, Pastora Gallego, Juan Gálvez Acebal, Jesús Rodríguez Baño; **Hospital Universitario Virgen del Rocío**, (Sevilla): Arístides de Alarcón, Emilio García, Juan Luis Haro, José Antonio Lepe, Francisco López, Rafael Luque; **Hospital San Pedro**, (Logroño): Luis Javier Alonso, José Manuel Azcona Gutiérrez, José Ramón Blanco, Lara García, José Antonio Oteo; **Hospital de la Santa Creu i Sant Pau**, (Barcelona): Natividad de Benito, Mercé Gurguí, Cristina Pacho, Roser Pericas, Guillem Pons; **Complejo Hospitalario Universitario de Santiago de Compostela**, (A Coruña): M. Álvarez, A. L. Fernández, Amparo Martínez, A. Prieto, Benito Regueiro, E. Tijeira, Marino Vega; **Hospital Santiago Apóstol**, (Vitoria): Andrés Canut Blasco, José Cordo Mollar, Juan Carlos Gainzarain Arana, Oscar García Uriarte, Alejandro Martín López, Zuriñe Ortiz de Zárate, José Antonio Urturi Matos; **Hospital SAS Línea de la Concepción**, (Cádiz): Mª Belén Nacle, Antonio Sánchez, Luis Vallejo; **Hospital Clínico Universitario Virgen de la Arrixaca** (Murcia): José Mª Arribas Leal, Elisa García Vázquez, Alicia Hernández Torres, Ana Blázquez, Gonzalo de la Morena Valenzuela; **Hospital de Txagorritxu**, (Vitoria): Ángel Alonso, Javier Aramburu, Felicitas Elena Calvo, Anai Moreno Rodríguez, Paola Tarabini-Castellani; **Hospital Virgen de la Salud,** (Toledo): Eva Heredero Gálvez, Carolina Maicas Bellido, Mª Antonia Sepúlveda; **Hospital Rafael Méndez,** (Lorca-Murcia): Eva Cascales Alcolea, Pilar Egea Serrano, José Joaquín Hernández Roca.

**Table S1:** Baseline characteristics of the total study population (n=2011).

| Variable | N= 2011 |
| --- | --- |
| Patient age, IQR (years) | 69 (57 - 76) |
| Male sex, n (%) | 1368 (68) |
| Comorbidities |  |
| Age adjusted Charlson comorbidity score, mean (SD) | 4.58 (2.6) |
| Chronic renal impairment, n (%) | 508 (25.2) |
| Diabetes mellitus, n (%) | 544 (27.1) |
| Cancer, n (%) | 314 (15.6) |
| Neurological disease, n (%) | 131 (6.5) |
| Predisposing conditions, n (%) |  |
| Pre-existing valve disease | 870 (43.3) |
| Congenital cardiac disease | 111 (5.5) |
| Previous endocarditis | 162 (8.1) |
| Intravenous drug abuse | 55 (2.7) |
| Echocardiography performed, n (%) |  |
| TTE | 1863 (92.6) |
| TEE | 1531 (76.1) |
| Site of infection, n (%) |  |
| Aortic | 952 (47.3) |
| Mitral | 894 (44.5) |
| Pulmonary | 29 (1.4) |
| Tricuspid | 114 (5.7) |
| Native | 1277 (63.5) |
| Prosthetic | 571 (28.4) |

IQR: Interquartile range; SD: Standard deviation; TTE: Transthoracic echocardiogram; TEE: Transesophageal echocardiography

**Table S2:** Clinical, microbiological and echocardiographic characteristics of HCM patients complicated by IE according to English and Spanish language case reports published between 1961 and 2014 (n=84).

| N | Age | Gender | NYHA | Wall thickness | Hypertrophy | ASM | LVOTO | Gradient | Affected valve | Predisposing factor | Infective agent | Surgery | Death | Year | Author |
| --- | --- | --- | --- | --- | --- | --- | --- | --- | --- | --- | --- | --- | --- | --- | --- |
| 1 | 37 | F | U | U | U | U | U | U | U | Dental procedure | Streptococcus viridans | U | No | 1961 | Boiteau et al. (w1) |
| 2 | 23 | F | U | U | U | U | U | U | Mitral | Abortion | Streptococcus viridans | U | No | 1961 | Boiteau et al. (w1) |
| 3 | 29 | M | I | U | U | U | Yes | 30 mmHg | U | U | Streptococcus viridans | No | No | 1966 | Linhart et al. (w2) |
| 4 | 34 | M | II | 30 mm | Concentric | U | Yes | 40 mmHg | Aortic | Dermatitis | Negative | No | Yes | 1967 | Nagle et al. (w3) |
| 5 | 39 | M | I | 39 mm | Septal | U | Yes | 127 mmHg | Mitral | U | Negative | No | Yes | 1968 | Vecht et al. (w4) |
| 6 | 49 | F | III | U | U | U | Yes | 67 mmHg | U | U | Streptococcus | No | Yes | 1968 | Vecht et al. (w4) |
| 7 | 59 | F | II | U | U | U | No | U | U | U | Staphylococcus albus | No | No | 1968 | Vecht et al. (w4) |
| 8 | 45 | M | II | U | U | U | Yes | U | Aortic | Cardiac catheterization | Streptococcus viridans | Yes | No | 1968 | Frank et al. (w5) |
| 9 | 61 | F | II | U | U | U | Yes | U | U | U | Streptococcus viridans | Yes | No | 1968 | Frank et al. (w5) |
| 10 | 43 | F | II | U | U | U | Yes | U | U | U | U | U | No | 1968 | Frank et al. (w5) |
| 11 | 20 | F | U | U | U | U | U | U | U | Dental procedure | Streptococcus faecalis | | No | 1969 | Epstein et al. (w6) |
| 12 | 39 | M | U | U | U | U | U | U | U | Cardiac catheterization | Corynebacterium sp | U | No | 1969 | Epstein et al. (w6) |
| 13 | 58 | M | I | U | U | U | No | U | U | U | Streptococcus viridans | No | No | 1971 | Cardelia et al. (w7) |
| 14 | 40 | F | I | U | U | U | No | 116 mmHg | U | Dental procedure | Streptococcus viridans | No | No | 1975 | Garrido Pintor et al. (w8) |
| 15 | 43 | M | I | 25 mm | Concentric | U | Yes | U | Aortic and Mitral | U | Enterococcus | U | Yes | 1975 | Wang et al. (w9) |
| 16 | 34 | M | I | U | U | U | Yes | 50 mmHg | Mitral | U | Staphylococcus aureus | Yes | No | 1977 | Huret et al. (w10) |
| 17 | 60 | F | I | U | U | U | U | U | U | Dental procedure | Streptococcus mutans | No | No | 1977 | Robbins et (w11) |
| 18 | 61 | M | II | U | U | Yes | U | U | U | U | Streptococcus mutans | U | No | 1977 | Robbins et al. (w11) |
| 19 | 53 | M | II | U | U | Yes | U | U | U | U | Listeria monocytogenes | No | No | 1978 | Pitcher et al. (w12) |
| 20 | 58 | F | I | 22 mm | Septal | Yes | Yes | U | Aortic and LVOT | U | Streptococcus pneumoniae | No | Yes | 1979 | LeJemtel et al. (w13) |
| 21 | 66 | F | I | U | U | Yes | Yes | U | Aortic, mital and LVOT | U | Enterococcus | Yes | Yes | 1979 | LeJemtel et al. (w13) |
| 22 | 64 | F | II | U | U | U | Yes | 105 mmHg | Aortic and Mitral | U | Staphylococcus aureus | Yes | Yes | 1979 | Greenland et al. (w14) |
| 23 | 63 | F | I | 20 mm | Septal | Yes | Yes | U | Mitral | Varicose ulcer | Staphylococcus aureus | No | Yes | 1981 | Bosch Gil et al. (w15) |
| 24 | 53 | F | III | 21 mm | Septal | Yes | Yes | 100 mmHg | Aortic | U | Streptococcus viridans | Yes | Yes | 1981 | Mensa Pueyo et al. (w16) |
| 25 | 45 | M | II | U | U |  | Yes | 90 mmHg | U | Miectomy | Streptococcus viridans | No | No | 1982 | Chagnac et al. (w17) |
| 26 | 40 | M | II | U | U | Yes | Yes | U | U | U | Streptococcus viridans | No | No | 1982 | Chagnac et al. (w17) |
| 27 | 63 | F | I | U | U | Yes | Yes | U | U | U | Enterococcus | No | No | 1982 | Chagnac et al. (w17) |
| 28 | 76 | M | II | U | U | U | Yes | U | Mitral | U | U | No | No | 1982 | Martinez Orozco et al. (w18) |
| 29 | 38 | M | I | U | U | U | Yes | U | U | U | Actinobacillus | Yes | No | 1983 | Ah Fat et al. (w19) |
| 30 | 57 | M | I | 19 mm | U | Yes | Yes | 125 mmHg | Mitral | Dermatitis | Streptococcus viridans | No | No | 1983 | Ovsyshcher et al. (w20) |
| 31 | 61 | M | I | U | Septal | U | U | U | U | IV catheterization | Staphylococcus epidermidis | No | Yes | 1983 | Bolivar et al. (w21) |
| 32 | 52 | M | I | U | U | U | Yes | U | Mitral | U | Candida tropicalis | Yes | Yes | 1984 | Malouf et al. (w22) |
| 33 | 70 | M | I | U | U | U | Yes | 70 mmHg | Mitral | U | Streptococcus viridans / Lactobacillus spp | Yes | No | 1989 | Stulz et al. (w23) |
| 34 | 51 | M | U | 2.2 (IVS/PW) | Septal | U | U | U | Mitral | U | Streptococcus faecalis | Yes | No | 1990 | Alessandri et al. (2) |
| 35 | 76 | M | U | 1.8 (IVS/PW) | U | Yes | Yes | 40 mmHg | Aortic and Mitral | U | Streptococcus coagulasa negativo | No | No | 1990 | Alessandri et al. (2) |
| 36 | 64 | M | U | 2.8 (IVS/PW) | Septal | Yes | Yes | 80 mmHg | Aortic and Mitral | Hemorroidectomy | Streptococcus viridans | No | No | 1990 | Alessandri et al. (2) |
| 37 | 48 | F | U | 2.2 (IVS/PW) | Septal | U | Yes | 45 mmHg | Mitral | U | Streptococcus mitis | No | No | 1990 | Alessandri et al. (2) |
| 38 | 56 | F | U | 2.3(IVS/PW) | Septal | Yes | Yes | 30 mmHg | Aortic and Mitral | U | Brucella | No | No | 1990 | Alessandri et al. (2) |
| 39 | 68 | M | U | 1.8 (IVS/PW) | Septal | Yes | Yes | 30 mmHg | Aórtica y Mitral | U | Streptococcus faecalis | Yes | No | 1990 | Alessandri et al. (2) |
| 40 | 67 | F | U | 2.3 (IVS/PW) | Septal | Yes | Yes | 80 mmHg | Mitral | Sinovitis | U | No | No | 1990 | Alessandri et al. (2) |
| 41 | 22 | F | I | 20 mm | Concentric | Yes | Yes | 71 mmHg | Mitral | U | U | Yes | No | 1991 | Anguita et al. (w24) |
| 42 | 11 | F | I | 30 mm | Septal | Yes | Yes | 62 mmHg | Aortic and Mitral | U | Streptococcus sanguis | No | U | 1992 | Chen et al. (w25) |
| 43 | 12 | M | II | 20 mm | Septal | Yes | Yes | 62 mmHg | Mitral | U | U | Yes | No | 1992 | Roberts et al. (w26) |
| 44 | 25 | M | II | U | Septal | U | No | U | U | U | U | U | No | 1992 | Roberts et al. (w26) |
| 45 | 43 | M | II | 17 mm | Septal | Yes | No | 26 mmHg | Mitral | U | U | Yes | No | 1992 | Roberts et al. (w26) |
| 46 | 46 | M | U | 29 mm | Septal | No | Yes | 45 mmHg | Mitral | Dental procedure | Cardiobacterium hominis | Yes | No | 1992 | Roberts et al. (w26) |
| 47 | 63 | M | III | 32 mm | Septal | Yes | Yes | 85 mmHg | Mitral | Dental procedure | U | Yes | No | 1992 | Roberts et al. (w26) |
| 48 | 64 | M | II | 26 mm | Septal | No | No | 5 mmHg | Mitral | Cardiac catheterization | Enterococcus | Yes | No | 1992 | Roberts et al. (w26) |
| 49 | 71 | M | II | U | Septal | Yes | No | U | Mitral | U | Streptococcus (alpha hemolytic) | Yes | Yes | 1992 | Roberts et al. (w26) |
| 50 | 51 | M | I | 20 mm | Septal | Yes | No | U | Aortic | U | U | Yes | No | 1992 | Roberts et al. (w26) |
| 51 | 52 | F | II | 26 mm | Septal | No | Yes | 78 mmHg | Aortic | U | U | Yes | No | 1992 | Roberts et al. (w26) |
| 52 | 54 | F | I | 24 mm | Septal | No | Yes | 70 mmHg | Aortic | Cardiac catheterization | Streptococcus (alpha hemolytic) | Yes | No | 1992 | Roberts et al. (w26) |
| 53 | 58 | M | II | U | Septal | No | Yes | 140 mmHg | Aortic and Mitral | IV drug abuse | Streptococcus (alpha hemolytic) | Yes | Yes | 1992 | Roberts et al. (w26) |
| 54 | 29 | F | I | U | U | U | Yes | U | U | Dental procedure | Gemella morbillorum | Yes | No | 1994 | Kerr et al. (w27) |
| 55 | 30 | M | I | U | U | Yes | Yes | U | U | U | Neisseria elongata | No | No | 1998 | Hofstad et al. (w28) |
| 56 | 52 | M | I | 22 mm | U | U | Yes | U | Mitral | U | U | No | No | 1999 | Spirito et al. (1) |
| 57 | 23 | M | II | 30 mm | U | U | Yes | U | Mitral | IV drug abuse | U | No | Yes | 1999 | Spirito et al. (1) |
| 58 | 26 | M | III | 27 mm | U | U | Yes | U | Mitral | Dental procedure | Streptococcus sanguis | Yes | No | 1999 | Spirito et al. (1) |
| 59 | 31 | F | IV | 17 mm | U | U | Yes | U | Aortic and Mitral | Dental procedure | Staphylococcus aureus | Yes | No | 1999 | Spirito et al. (1) |
| 60 | 38 | M | III | 26 mm | U | U | Yes | U | Mitral | Dental procedure | Enterococcus faecalis | No | No | 1999 | Spirito et al. (1) |
| 61 | 49 | M | IV | 20 mm | U | U | Yes | U | Mitral | Dental procedure | U | Yes | No | 1999 | Spirito et al. (1) |
| 62 | 42 | M | II | 26 mm | U | U | Yes | U | Aortic and Mitral | U | Staphylococcus epidermidis | No | No | 1999 | Spirito et al. (1) |
| 63 | 48 | F | II | 28 mm | U | U | Yes | U | Mitral | U | Streprococcus viridans | No | No | 1999 | Spirito et al. (1) |
| 64 | 16 | M | III | 17 mm | U | U | Yes | U | Aortic and Mitral | U | Staphylococcus aureus | Yes | No | 1999 | Spirito et al. (1) |
| 65 | 62 | F | II | 26 mm | U | U | Yes | U | Mitral | U | U | No | No | 1999 | Spirito et al. (1) |
| 66 | 59 | M | U | U | Septal | Yes | Yes | 50 mmHg | Mitral | U | Staphylococcus epidermidis | Yes | No | 2002 | Decastro et al. (w29) |
| 67 | 42 | M | U | U | U | Yes | Yes | 65 mmHg | Aortic and Mitral | Dental procedure | Staphylococcus epidermidis | Yes | No | 2002 | Decastro et al. (w29) |
| 68 | 35 | F | U | U | U | Yes | Yes | 80 mmHg | Aortic | U | Staphylococcus aureus | No | Yes | 2002 | Decastro et al. (w29) |
| 69 | 27 | M | I | 24 mm | Septal | Yes | Yes | 36 mmHg | Mitral | Dental procedure | Staphylococcus aureus | Yes | No | 2002 | Morgan-Hughes et al. (w30) |
| 70 | 29 | M | I | U | U | Yes | Yes | 100 mmHg | Mitral | U | Streptococcus viridans | Yes | U | 2004 | Inoue et al. (w31) |
| 71 | 27 | F | I | 21 mm | Septal | U | No |  | Apical septum | IV drug abuse | Listeria monocytogenes | No | No | 2006 | Bosch et al. (w32) |
| 72 | 46 | F | I | 32 mm | Septal | Yes | Yes | 45 mmHg | Aortic and septum | U | Streptococcus viridans | Yes | No | 2006 | Pachirat et al. (w33) |
| 73 | 43 | M | I | 18 mm | Septal | Yes | Yes | 64 mmHg | Mitral | Suboptimal oral health | Streptococcus viridans | Yes | No | 2006 | Louahabi et al. (w34) |
| 74 | 53 | M | U | U | U | U | No |  | Mitral | U | Staphylococcus hominis | No | No | 2007 | Cunha et al (w35) |
| 75 | 75 | F | II | U | Septal | Yes | Yes | 50 mmHg | Mitral and septum | Septal ablation | U | No | Yes | 2008 | Zemanek et al (w36) |
| 76 | 52 | F | U | 14 mm | U | U | No | 20.4 mmHg | Aortic and Mitral | U | Streptococcus viridans | Yes | No | 2009 | Yuan et al. (w37) |
| 77 | 74 | F | II | U | U | U | Yes |  | Mitral | U | Candida kefyr | No | No | 2010 | Chopra et al. (w38) |
| 78 | 75 | F | IV | U | Septal | U | Yes | 80 mmHg | Mitral and septum | Septal ablation | U | No | Yes | 2010 | Curiel Balsera et al. (w39) |
| 79 | 31 | M | I | U | Septal | U | Yes |  | Septum and LVOT | IV drug abuse | Staphylococcus aureus | No | Yes | 2010 | Fyfe et al. (w40) |
| 80 | 52 | M | III | 16 mm | Septal | Yes | Yes | 108 mmHg | Septum | Dental abscess and septal ablation | Staphylococcus aureus | No | No | 2011 | Liang et al. (w41) |
| 81 | 59 | M | I | U | U | U | Yes | 35 mmHg | Mitral | Dental procedure | Actinobacillus endocarditis | Yes | No | 2012 | Jorge et al. (w42) |
| 82 | 62 | M | I | 18 mm | Concentric | Yes | Yes | 58 mmHg | Aortic and Mitral | IV catheterization | Negative | Yes |  | 2012 | Katayama el al. (w43) |
| 83 | 17 | F | I | 22 mm | Septal | U | No | 100 mmHg | Aortic | Dental procedure | Streptococcus sanguis-gordonii | No | No | 2013 | Noel et al. (w44) |
| 84 | 37 | F | U | 17 mm | Septal | Yes | Yes | 130 mmHg | Mitral | Dental procedure | Negative | No | Yes | 2013 | Guler et al. (w45) |

IV: intravenous. LVOT: Left ventricular outflow tract. M: Male. F: Female. U: Unknown.

**Table S3:** Baseline characteristics of patients with non-device infective endocarditis (n=1807)

| Variable | N= 1807 |
| --- | --- |
| Patient age, IQR (years) | 68 (56 - 76) |
| Male sex, n (%) | 1213 (67.1) |
| Comorbidities |  |
| Age adjusted Charlson comorbidity score, mean (SD) | 4.54 (2,6) |
| Chronic renal impairment, n (%) | 441 (24.4) |
| Diabetes mellitus, n (%) | 479 (26.5) |
| Cancer, n (%) | 298 (16.5) |
| Neurological disease, n (%) | 120 (6.6) |
| Predisposing conditions, n (%) |  |
| Pre-existing valve disease | 831 (46.0) |
| Congenital cardiac disease | 103 (5.7) |
| Previous endocarditis | 144 (8.0) |
| Intravenous drug abuse | 55 (3.0) |
| Echocardiography performed, n (%) |  |
| TTE | 1685 (93.2) |
| TEE | 1373 (76.0) |
| Site of infection, n (%) |  |
| Aortic | 943 (52.3) |
| Mitral | 885 (49) |
| Pulmonary | 28 (1.5) |
| Tricuspid | 97 (5.4) |
| Native | 1246 (69.0) |
| Prosthetic | 559 (31) |

IQR: Interquartile range; SD: Standard deviation; TTE: Transthoracic echocardiogram; TEE: Transesophageal echocardiography.

**Supplemental references**

W1 Boiteau GM, Allenstein BJ. Hypertrophic subaortic stenosis: Clinical and hemodynamic studies with special reference to pulse contour measurement. *Am J Cardiol.* 1961;8:614-623.

W2. Linhart JW, Taylor WJ. Bacterial endocarditis in a patient with idiopathic hypertrophic subaortic stenosis. *Circulation.* 1966;34:595-596.

W3. Nagle JP. Idiopathic hypertrophic subaortic stenosis and bacterial endocarditis. *JAMA*. 1967;200:643-645.

W4. Vecht RJ, Oakley CM. Infective endocarditis in three patients with hypertrophic obstructive cardiomyopathy. *Br Med J.* 1968;2:455-459.

W5. Frank S, Braunwald E. Idiopathic hypertrophic subaortic stenosis. Clinical analysis of 126 patients with emphasis on the natural history. *Circulation.* 1968;37:759-788.

W6. Epstein EJ, Coulshed N. Bacterial endocarditis in idiopathic hypertrophic subaortic stenosis. *Cardiologia.* 1969;54:30-36.

W7. Cardelia JU, Befeler B, Hildner FJ, Samet P. Hypertrophic subaortic stenosis complicated by aortic insufficiency and subacute bacterial endocarditis. *Am Heart J.* 1971;81: 543–547.

W8. Garrido Pintor A, Flores Pedauye A, Peris Monfort M, Hernández Martínez M, Palacios Montilla V, Algarra Vidal FJ. Bacterial endocarditis as a complication of dynamic hypertrophic subaortic stenosis. Report of a case. *Rev Esp Cardiol.* 1975;29:267-271.

W9. Wang K, Gobel FL, Gleason DF. Bacterial endocarditis in idiopathic hypertrophic subaortic stenosis. *Am Heart J.* 1975;89:359-365

W10. Huret JF, Jandin M, Rigaud M, et al. Endocarditis due to staphylococcus aureus during cardiomyopathy. *Arch Mal Coeur Vaiss.* 1977;70:1227-1232.

W11. Robbins N, Szilagyi G, Tanowitz HB, Luftschein S, Baum SG. Infective endocarditis caused by Streptococcus mutans. A complication of idiopathic hypertrophic subaortic stenosis. *Arch Intern Med.* 1977;137:1171-1174.

W12. Pitcher D, Mary D. Listeria monocytogenes endocarditis in hypertrophic cardiomyopathy Haemarthrosis of the knee presenting as ankle bruising. *Br Med J.* 1978;15:961.

W13. LeJemtel TH, Factor SM, Koenigsberg M, O'Reilly M, Frater R, Sonnenblick EH. Mural vegetations at the site of endocardial trauma in infective endocarditis complicating idiopathic hypertrophic subaortic stenosis. *Am J Cardiol.* 1979;44:569-574.

W14. Greenland P, Murphy GW. Acute Valvular Insufficiency Complicating Hypertrophic Obstructive Cardiomyopathy. *Chest.* 1979;72:182-183.

W15. Bosch Gil J, Arnau de Bolós JM, Pigrau Serrallach C. Bacterial endocarditis and its relationships with obstructive hypertrophic cardiomyopathy and hepatic cirrhosis. *Med Clin (Barc).* 1981;76:367-369.

W16. Mensa Pueyo J, Shahin A, Martinez Benazet J, Moreno Camacho A, Garcia San Miguel J. Infectious endocarditis in idiopathic hypertrophic subaortic stenosis. Report of a case . *Med Clin (Barc).* 1981;76:129-131.

W17. Chagnac A, Rudniki C, Loebel H, Zahavi I. Infectious endocarditis in idiopathic hypertrophic subaortic stenosis: report of three cases and review of the literature. *Chest.* 1982;81:346-349.

W18. Martínez-Orozco F, Ancochea L, Valls V, Ingelmo M, Balcells-Gorina A. Subacute infectious endocarditis in an elderly patient suffering from obstructive cardiomyopathy. *Rev Clin Esp.* 1982; 166:245-247.

W19. Ah Fat LN, Patel BR, Pickens S. Actinobacillus actinomycetemcomitans endocarditis in hypertrophic obstructive cardiomyopathy. *J Infect.* 1983;6:81-84.

W20. Ovsyshcher IA, Zimlichman R. Infective endocarditis in hypertrophic cardiomyopathy secondary to amiodarone treatment. *Chest.* 1983;83:833.

W21. Bolivar R. Asymmetric septal hypertrophy in the differential diagnosis of endocarditis. *Arch Intern Med.* 1983;143:2024-2025.

W22. Malouf J, Nasrallah A, Daghir I, Harake M, Mufarrij A. Candida tropicalis endocarditis in idiopathic hypertrophic subaortic stenosis. *Chest*. 1984; 86:508.

W23. Stulz P, Zimmerli W, Mihatsch J, Grädel E. Recurrent infective endocarditis in idiopathic hypertrophic subaortic stenosis. *Thorac Cardiovasc Surg.* 1989;37:99-102.

W24. Anguita M, Romo E, Viñals M, et al. The management by medical treatment of an intracranial mycotic aneurysm in a patient with infectious endocarditis with negative blood cultures and hypertrophic myocardiopathy. *Rev Esp Cardiol.* 1991;44:556-559.

W25. Chen MR. Infective endocarditis in hypertrophic obstructive cardiomyopathy. *J Clin Ultrasound.* 1992;20:612-614.

W26. Roberts WC, Kishel JC, McIntosh CL, Cannon RO, Maron BJ. Severe mitral or aortic valve regurgitation, or both, requiring valve replacement for infective endocarditis complicating hypertrophic cardiomyopathy. *J Am Coll* *Cardiol.* 1992; 19:365-371.

W27. Kerr JR, Webb CH, McGimpsey JG, Campbell NP. Infective endocarditis due to Gemella morbillorum complicating hypertrophic obstructive cardiomyopathy. *Ulster Med J.* 1994;63:108-110.

W28. Hofstad T, Hope O, Falsen E. Septicaemia with Neisseria elongata ssp. nitroreducens in a patient with hypertrophic obstructive cardiomyopathia. *Scand J Infect Dis.* 1998;30:200-201.

W29. De Castro S, Adorisio R, Pelliccia A, Papetti F, Fedele F, Pandian N. Perforated aneurysms of left side valves during active infective endocarditis complicating hypertrophic obstructive cardiomyopathy. *Eur J Echocardiogr.* 2002;3:100-102.

W30. Morgan-Hughes G, Motwani J. Mitral valve endocarditis in hypertrophic cardiomyopathy: case report and literature review. *Heart.* 2002;87:e8.

W31. Inoue T, Shinohara T, Saga T. Surgical treatment of infective endocarditis complicated by intracranial hemorrhage in a patient with hypertrophic obstructive cardiomyopathy. *Can J Cardiol.* 2004;20:645-653.

W32. Bosch MJ, Fácila L, Nuñez J, et al. A non-valvular infective endocarditis in an HIV patient with myocardiopathy. *Int J Cardiol.* 2006;107:115-116.

W33. Pachirat O, Klungboonkrong V, Tantisirin C. Infective endocarditis in hypertrophic cardiomyopathy- mural and aortic valve vegetations: a case report. *J Med Assoc Thai.* 2006 ;89:522-526.

W34. Louahabi T, Drighil A, Habbal R, Azzouzi L. Infective endocarditis complicating hypertrophic obstructive cardiomyopathy. *Eur J Echocardiogr.* 2006;7:468-470.

W35. Cunha BA, Esrick MD, Larusso M. Staphylococcus hominis native mitral valve bacterial endocarditis (SBE) in a patient with hypertrophic obstructive cardiomyopathy. *Heart Lung.* 2007;36:380-382.

W36. Zemanek D, Veselka J, Chmelova R. Infective endocarditis after alcohol septal ablation for obstructive hypertrophic cardiomyopathy. *Int Heart J*. 2008;49:371–375

W37. Yuan SM, Demesthenous E, Coman V. Long QT syndrome in extensive infective endocarditis complicating hypertrophic obstructive cardiomyopathy. *Kardiol Pol.* 2009;67:53-57.

W38. Chopra T, Bhargava A, Kumar S, Chopra A. Candida kefyr endocarditis in a patient with hypertrophic case report. *Am J Med Sci.* 2010;339:188-9.

W39. Curiel Balsera E, Cano Nieto J, Muñoz Bono J. Infective endocarditis after alcohol septal ablation for obstructive hypertrophic cardiomyopathy. *Minerva Cardioangiol.* 2010;58:422-423.

W40. Fyfe B, Ianosi-Irimie M, Motavalli L. Infective endocarditis complicating hypertrophic obstructive cardiomyopathy: an unusual mural pattern. *Cardiovasc. Pathol.* 2010;19:e5-7.

W41. Liang M, Pasupati S, Jogia D. Post-Transcoronary Ethanol Septal Ablation (TESA) Infective Endocarditis complicated by a ventricular septal defect. *J Invasive Cardiol.* 2011;23:4-8.

W42. Jorge VC, Araújo AC, Grilo A, et al. Actinobacillus endocarditis associated with hypertrophic cardiomyopathy. *BMJ Case Rep.* 2012; doi: 10.1136/bcr.04. 2011.4140.

W43. Katayama T, Tsuruya Y, Ishikawa S. Complete Atrioventricular Block and Infective Endocarditis in a Patient with Hypertrophic Obstructive Cardiomyopathy. *Intern Med.* 2012;51:749-753.

W44. Noel N, Naheed Z. Hypertrophic cardiomyopathy: role of current recommendations by the american heart association for infective endocarditis. *Pediatr Cardiol.*2013;34:709-11.

W45. Guler A, Aung SM, Cakal B, Karabay CY, Guler Y, Kirma C. Infective endocarditis complicating hypertrophic obstructive cardiomyopathy: is antibiotic prophylaxis really unnecessary? *Curr Cardiol Rev.* 2013;9:308-9.
